# Genome-wide identification and adaptive evolution of CesA/Csl superfamily among species with different life forms in Orchidaceae

**DOI:** 10.3389/fpls.2022.994679

**Published:** 2022-09-29

**Authors:** Jingjing Wang, Jing Li, Wei Lin, Ban Deng, Lixian Lin, Xuanrui Lv, Qilin Hu, Kunpeng Liu, Mahpara Fatima, Bizhu He, Dongliang Qiu, Xiaokai Ma

**Affiliations:** ^1^Center for Genomics and Biotechnology, School of Future Technology, Haixia Institute of Science and Technology, Fujian Agriculture and Forestry University, Fuzhou, China; ^2^College of Horticulture, Fujian Agriculture and Forestry University, Fuzhou, China; ^3^College of Forestry, Fujian Agriculture and Forestry University, Fuzhou, China; ^4^College of Life Sciences, Fujian Agriculture and Forestry University, Fuzhou, China; ^5^Key Laboratory of Orchid Conservation and Utilization of National Forestry and Grassland Administration at College of Landscape Architecture, Fujian Agriculture and Forestry University, Fuzhou, China

**Keywords:** orchids, epiphyte, terrestrial, saprophytic, CesA/Csl, life-history, expression patterns

## Abstract

Orchidaceae, with more than 25,000 species, is one of the largest flowering plant families that can successfully colonize wide ecological niches, such as land, trees, or rocks, and its members are divided into epiphytic, terrestrial, and saprophytic types according to their life forms. Cellulose synthase (*CesA*) and cellulose synthase-like (*Csl*) genes are key regulators in the synthesis of plant cell wall polysaccharides, which play an important role in the adaptation of orchids to resist abiotic stresses, such as drought and cold. In this study, nine whole-genome sequenced orchid species with three types of life forms were selected; the CesA/Csl gene family was identified; the evolutionary roles and expression patterns of CesA/Csl genes adapted to different life forms and abiotic stresses were investigated. The CesA/Csl genes of nine orchid species were divided into eight subfamilies: CesA and CslA/B/C/D/E/G/H, among which the CslD subfamily had the highest number of genes, followed by CesA, whereas CslB subfamily had the least number of genes. Expansion of the CesA/Csl gene family in orchids mainly occurred in the CslD and CslF subfamilies. Conserved domain analysis revealed that eight subfamilies were conserved with variations in orchids. In total, 17 pairs of CesA/Csl homologous genes underwent positive selection, of which 86%, 14%, and none belonged to the epiphytic, terrestrial, and saprophytic orchids, respectively. The inter-species collinearity analysis showed that the CslD genes expanded in epiphytic orchids. Compared with terrestrial and saprophytic orchids, epiphytic orchids experienced greater strength of positive selection, with expansion events mostly related to the CslD subfamily, which might have resulted in strong adaptability to stress in epiphytes. Experiments on stem expression changes under abiotic stress showed that the CslA might be a key subfamily in response to drought stress for orchids with different life forms, whereas the CslD might be a key subfamily in epiphytic and saprophytic orchids to adapt to freezing stress. This study provides the basic knowledge for the further systematic study of the adaptive evolution of the CesA/Csl superfamily in angiosperms with different life forms, and research on orchid-specific functional genes related to life-history trait evolution.

## Introduction

Stored carbohydrates serve as a carbon and energy resource for land plant growth and against adverse and favorable conditions (Ranwala and Miller, 2008), and have contributed to drought resistance, frost resistance, salt tolerance, and penetration (Mattana et al., 2005; Rosa et al., 2009; Zhang et al., 2016). The cell wall, an important part of plant cells, consists of a basic skeleton of polysaccharides that constitutes the plant’s main carbon sink, which is ultimately maintained by the plant’s ability to fix carbon dioxide through photosynthesis (Lampugnani et al., 2018). Plants overcome the high intracellular osmotic pressure to a certain extent by assembling some photosynthetic carbohydrate products into cell wall polysaccharides, thereby increasing the strength and flexibility of terrestrial plant cell walls, enabling individual cells to withstand enormous swelling pressures, preventing the rupture of membrane and the structure that controls cell growth, and enabling plants to better cope with environmental stresses, such as drought, freezing, and osmosis (Sarkar et al., 2009). Therefore, the synthesis of plant cell wall polysaccharides plays an important role in the growth and adaptive evolution of plants with different life forms living in extreme or harsh natural habitats.

Studies have shown that mannan is one of the main components of the cell wall polysaccharide in plants. For example, mannan accounts for 58.3% of the dry weight of the crude polysaccharide of the orchid *Dendrobium officinale* (Xing et al., 2015). The key enzymes involved in the biosynthesis of mannan belong to the cellulose synthase (CesA) family (Lerouxel et al., 2006). The CesA family can be subdivided into one cellulose synthase family (CesA) and eight cellulose synthases-like (CslA-CslH), in which each subfamily is involved in regulating different life processes that are involved in cell wall polysaccharide synthesis (Richmond and Somerville, 2000; Suzuki et al., 2006; Little et al., 2018). The main function of CesA is to participate in primary and secondary cell wall synthesis (Hamann et al., 2004; Farrokhi et al., 2006). CslA mainly encodes β-1,4-mannan synthase (Yin et al., 2009). CslC is involved in catalyzing the formation of the xyloglucan skeleton (Kim et al., 2020). CslD is involved in the synthesis of cell wall polysaccharides, mainly xylan and galacturonan (Verhertbruggen et al., 2011; Yang et al., 2020). CslF and CslH mediate β-(1,3;1,4)-D-glucan synthesis (Burton et al., 2006; Doblin et al., 2009). The CslG gene family is generally considered to be involved in the synthesis of cell wall polysaccharides (Richmond and Somerville, 2000). However, the biological functions of CslB and CslE are unclear (Richmond and Somerville, 2000).

Orchidaceae is one of the largest and most widely distributed families of flowering plants with more than 25,000 species, accounting for approximately 10% of flowering plant species (Leitch et al., 2009). Orchids have unique flower morphologies and extraordinary lifestyle diversity and are distributed in almost every habitat on the Earth (Roberts and Dixon, 2008; Givnish et al., 2015, 2016). Orchids can successfully colonize terrestrial, epiphytic, or lithophytic ecological niches, and attach to trees or rocks; they are mainly divided into epiphytic, saprophytic, and terrestrial types, and utilize crassulacean acid metabolism (CAM) for growth under different environmental conditions (Cai et al., 2015; Zhang et al., 2016). Several studies showed that orchids especially those (e.g., *Dendrobium* genus) with fleshy stems are rich in various types of active polysaccharides in the cell wall, which are related to drought or cold stress adaptation to different environmental conditions (Xing et al., 2015; Jin et al., 2016; Zhang et al., 2016; Wan et al., 2018).

Over the past two decades, the number of sequenced species has increased exponentially with advances in genome-sequencing technology and genome-assembly algorithms. Sequences of more than 1,000 plant genomes have been published, representing more than 790 different species with highly diverse life histories (Marks et al., 2021; Sun et al., 2021). Since the release of the first orchid genome of *Phalaenopsis equestris* (Cai et al., 2015), other orchids, such as *D. officinale* (Zhang et al., 2016), *D. huoshanense* (Han et al., 2020), *D. chrysotoxum* (Zhang et al., 2021), *Apostasia shenzhenica* (Zhang et al., 2017), *Cymbidium ensifolium* (Ai et al., 2021), *P. aphrodite* (Chao et al., 2018), *Vanilla planifolia* (Hasing et al., 2020), and *Gastrodia elata* (Xu et al., 2021) have been sequenced. Among the above nine orchid species, *D. officinale*, *D. huoshanense*, *D. chrysotoxum*, *P. equestris*, and *P. aphrodite* are epiphytic; *C. ensifolium*, *V. planifolia*, and *A. shenzhenica* are terrestrial; and *G. elata* is saprophytic. These species differ in the composition of the cell wall membrane, which synthesizes various types of active polysaccharides by the expression and regulation of related CesA/Csl genes, while their life forms are divergent *via* adapting to their specific local environmental conditions (Zhang et al., 2016; Lan et al., 2019; Gao et al., 2020; Idris et al., 2021; Xi et al., 2021).

To date, few studies have conducted evolutionary studies of the gene families related to polysaccharide synthesis in plant congeners with different growth types or life forms regarding their adaptation to environmental stress. However, the available whole-genome sequences of orchids with different life forms provided a unique opportunity to identify and study the adaptive evolution of the CesA/Csl superfamily, which is involved in polysaccharide synthesis. Therefore, we used nine whole-genomes sequenced orchid species with three types of life forms (terrestrial, epiphytic, and saprophytic) and the genomes of *Oryza sativa* and *Arabidopsis thaliana* to compare the patterns of gene family divergence and construct the evolutionary pathways of the members of the CesA/Csl superfamily among the three types of life forms. Furthermore, the gene structure related to protein motifs and conserved domains, evolutionary selection pressures, gene collinearity among species were systematically analyzed, and the expression profiles detection and qRT-PCR validation in stem tissues under abiotic stress were conducted, to comprehensively study the adaptive evolution of CesA/Csl superfamily in species with different life forms in the orchid family.

## Materials and methods

### Data sources

Whole-genome and protein sequences were downloaded from the China National Gene Bank (CNGB),^1^ National Center for Biotechnology Information (NCBI),^2^ and RGAP^3^ databases for the studied Orchidaceae species (*D. officinale, D. huoshanense, D. chrysotoxum, P. aphrodite, P. equestris, C. ensifolium, G. elata, V. planifolia*, and *A. shenzhenic*) and *O. sativa* (Supplementary Table 1). *Dendrobium officinale* annotation was generated using the GETA annotation pipeline (Supplementary Table 1). The corresponding protein sequences of CesA and Csl were obtained from the Arabidopsis information resource (TAIR) database.^4^ The RNA-seq raw reads of the 10 species were downloaded from the NCBI sequence read archive (SRA) database^5^ (Supplementary Table 2).

### Identification of CesA/Csl gene family members

Two methods were applied to identify the gene members in CesA/Csl superfamily in orchid genomes. First, the hidden Markov model (HMM) profiles of two domains Cellulose_synt (PF03552) and zf-UDP (PF14569) retrieved from the Pfam database were used against the protein database of 11 species using HMMER version 3.0, with a threshold of E < 1e-10 (Sun and Buhler, 2007; Finn et al., 2011). Second, the BLASTP (Mahram and Herbordt, 2015) search was performed using Ces/Csl protein sequences of *A. thaliana* and *O. sativa* as queries against the protein database of 11 species with the threshold E < 1e-10, and sequences with identity > 50% were retained. We extracted the common protein sequences identified by both hmmsearch and BLASTP searches and submitted them to the PfamScan website^6^ for domain alignment. Genes with E < 1e-20 and containing PF03552 and PF14569 domains were finally defined as members of the CesA/Csl family (Supplementary Table 3).

### Phylogenetic tree construction

The protein sequences of CesA/Csl genes of the 11 species were used for multiple sequence alignment using the MUSCLE software (Edgar, 2004) with default parameters. The BMGE software (Criscuolo and Gribaldo, 2010) was used to filter non-conserved sequences before tree construction. The phylogenetic tree under the optimal model was constructed using IQtree2 (Minh et al., 2020) with the parameter -m MFP. According to the clustering relationships with *A. thaliana* and *O. sativa*, the CesA/Csl gene family members in the nine orchid species were classified into subfamilies, and the CesA/Csl family members of each species were renamed according to the classification of subfamilies.

### Protein motif distribution and conserved domains

We used the MEME software^7^ to predict conserved motifs in the protein sequences of each subfamily. The maximum number of motifs was 20 and the other parameters were default values. Using NCBI conserved domains database,^8^ the “Pfam–18271 PSSMs” database was selected for conserved domain search for each subfamily. Additionally, we used TBtools (Chen et al., 2020a) to display the distribution and regularity of the protein motifs and conserved domains corresponding to each subfamily.

### Selection pressure analysis and interspecies collinearity of CesA/Csl genes

After pairwise matching of the coding sequences (CDS) of the corresponding subfamilies of nine orchids, the KaKs_Calculator (Zhang, 2022) software was used to calculate the ratio of non-synonymous (*Ka*) and synonymous substitution rates (*Ks*) of CesA/Csl genes under different selection pressures; *Ka*/*Ks* > 1 represents positive selection, *Ka*/*Ks* < 1 denotes negative selection, and *Ka*/*Ks* = 1 indicates neutral evolution (Yadav et al., 2015). In addition, McscanX (Wang et al., 2012) was used to calculate inter-species CesA/Csl gene collinearity with a BLASTP threshold E < 1e-5. TBtools (Chen et al., 2020a) was used to display the collinearity of the CesA/Csl genes of *A. shenzhenic* with other orchid species to determine the contraction and expansion of the gene families.

### Transcriptome analysis of CesA/Csl genes in nine orchid species

First, we used fastq-dump (Edwards and Edwards, 2019) to convert RNA-seq raw data (Supplementary Table 2) into fastq format and then used Trimmomatic (Bolger et al., 2014) for quality control of the sequences. The parameters were set as follows: ILLUMINACLIP: TruSeq3-SE:2:30:10, LEADING:3, TRAILING:3, SLIDINGWINDOW:4:15, and MINLEN:36. Thereafter, trim_galore^9^ was used to remove low-quality reads and linkers with the following parameters: –length, 75; quality, 25; stringency, 5. After indexing the genome and transcripts, the data were aligned to the reference genome using HISAT2 (Guo et al., 2022). The featureCounts software (Liao et al., 2014) was used to calculate the count value of the transcriptome data that matched the genome data. We extracted the length of the gene corresponding to the exon in the corresponding annotation file of each species and associated the total number of reads, count value, and gene length file on the mapping to obtain the FPKM gene expression matrix corresponding to each species. Finally, CesA/Csl gene expression levels in each species were represented in a heatmap to summarize the differences.

### Real-time quantitative polymerase chain reaction

*Dendrobium officinale*, *C. ensifolium*, and *G. elata* were selected as representative orchid species for the three life forms (epiphytic, terrestrial, and saprophytic). After treatment with drought (20 days) and freezing (0 and 20°C as control), the stem parts were collected and immediately frozen in liquid nitrogen. Total RNA was extracted from each sample using a TIANGEN polysaccharide and polyphenol plant total RNA extraction kit [Tiangen Biotech (Beijing) Co., Ltd.], and cDNA was synthesized using a TaKaRa PrimeScript™ RT reagent kit with a gDNA Eraser (Perfect Real Time) kit. Primers were designed using the Primer Premier 5 software (Lalitha, 2000; Supplementary Table 5). Real-time quantitative reverse transcription polymerase chain reaction (RT-qPCR) was performed using the TB Green Premix Ex Taq™ II kit (Tli RNase H Plus) and the CFX connect™ Real-Time PCR Detection System. The reaction mixture (20 μl) included 10 μl of TB Green Premix Ex Taq II (Tli RNaseH Plus) (2X), 0.5 μl of PCR Forward Primer (10 μM), 0.5 μl of PCR Reverse Primer (10 μM), 2 μl of fivefold diluted cDNA template, and 7 μl of ddH_2_O. The reactions were performed according to the following cycling profile: Pre-denaturation at 95°C for 3 min; denaturation at 95°C for 10 s, annealing at 60°C for 30 s, and 40 cycles. Then, the temperature was slowly increased from 65 to 95°C for melting curve analysis. Three technical replicates were performed for each sample. Gene expression levels were calculated using the 2-ΔΔC^T^ method as previously described (Livak and Schmittgen, 2001). We used the GraphPad prism8 software (Swift, 1997) to test the normal distribution of the data. Pairwise *t*-tests were performed to determine whether the differences in expression were significant.

## Results

### Identification of CesA/Csl gene family members in orchids

To identify the CesA/Csl genes extensively, we explored nine orchid genomes using the HMM profile of two domains [Cellulose_synt (PF03552) and zf-UDP (PF14569)] and BLASTP searches using 40 CesA/Csl protein sequences of *A. thaliana* and 45 CesA/Csl protein sequences of *O. sativa* as the query. A total of 349 CesA/Csl were identified in nine orchid species, with 51, 40, 40, 32, 36, 48, 24, 45, and 33 CesA/Csl genes in *D. officinale*, *D. huoshanense*, *D. chrysotoxum*, *P. aphrodite*, *P. equestris*, *C. ensifolium*, *G. elata*, *V. planifolia*, and *A. shenzhenica*, respectively (Table 1). While 45 and 40 CesA/Csl genes were identified in *O. sativa* and *A. thaliana*, separately. Among all the species, saprophytic orchid *G. elata* had the lowest (24) and epiphytic orchid *D. officinale* had the highest (51) number of CesA/Csl genes. The numbers of CesA/Csl genes in *D. officinale*, *C. ensifolium*, *V. planifolia*, and *O. sativa* were higher than that in *A. thaliana*, while the numbers of CesA/Csl genes in *P. aphrodite, P. equestris, A. shenzhenica*, and *G. elata* were less than that in *A. thaliana*, and the numbers of CesA/Csl genes in *D. huoshanense* and *D. chrysotoxum* were the same as that in *A. thaliana*. Within each type of the epiphytic, terrestrial, and saprophytic orchids, *D. officinale* (51), *C. ensifolium* (48), and *G. elata* (24) had the highest number of CesA/Csl genes, respectively. The results showed that the number distribution of CesA/Csl genes in the nine orchid species is quite varied and divergent within and among different life forms.

**TABLE 1:** Members of CesA/Csl gene family in nine orchid species, *A. thaliana*, and *O. sativa.*

Species	Life form	Subfamily									
		CesA	CslA	CslB	CslC	CslD	CslE	CslF	CslG	CslH	Sum
*D. officinale*	Epiphyte	9	10	/	5	15	4	/	7	1	51
*D. chrysotoxum*	Epiphyte	12	8	/	6	8	1	/	4	1	40
*D. huoshanense*	Epiphyte	10	7	/	4	16	2	/	1	/	40
*P. aphrodite*	Epiphyte	8	6	/	3	9	3	/	1	2	32
*P. equestris*	Epiphyte	11	6	1	3	10	2	/	1	2	36
*C. ensifolium*	Terrestrial	10	7	4	5	9	4	/	6	3	48
*V. planifolia*	Terrestrial	15	6	2	4	14	3	/	/	1	45
*A. shenzhenica*	Terrestrial	9	9	/	4	7	1	/	2	1	33
*G. elata*	Saprophytic	9	6	/	2	6	1	/	/	/	24
*A. thaliana*	Terrestrial	10	9	6	5	6	1	/	3	/	40
*O. sativa*	Terrestrial	11	9	/	6	5	3	8	/	3	45

### Phylogenetic analysis and subfamilies classification

**FIGURE 1:**
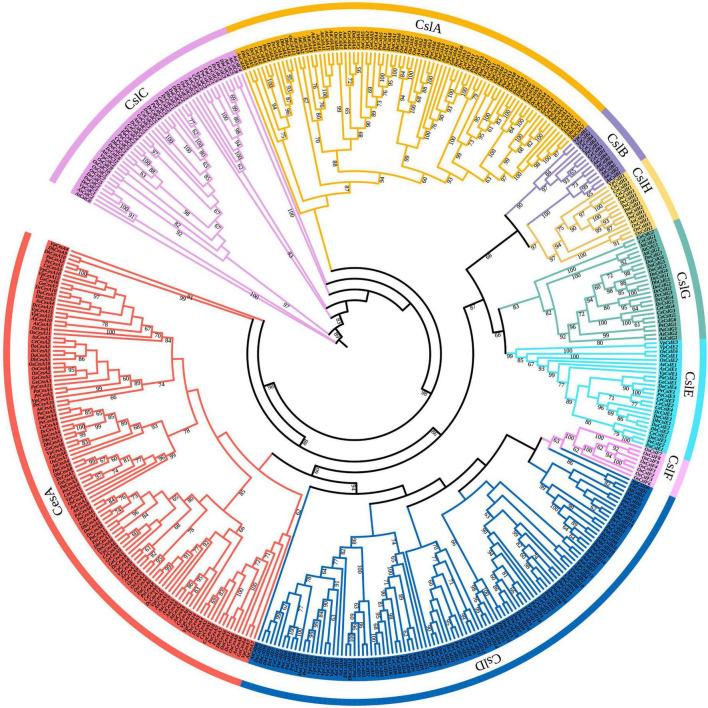
Phylogenetic tree of CesA/Csl superfamily for nine orchid species as well as *A. thaliana* and *O. sativa*. CesA, CslA, CslB, CslC, CslD, CslE, CslF, CslG, and CslH are nine subfamilies. The different colored blocks represent the distribution of different subfamilies. The numbers on the branches represent the bootstrap value (60–100).

To classify and detect the evolutionary relationship among CesA/Csl genes in Orchidaceae, a phylogenetic tree of CesA/Csl genes among nine orchids species was constructed (Figure 1). Based on the clustering relationships with *A. thaliana* and *O. sativa*, the typologies of the phylogenetic tree showed that CesA/Csl genes were classified into nine subfamilies: CesA, CslA, CslB, CslC, CslD, CslE, CslF, CslG, and CslH (Table 1). A comparison of subfamilies among different orchid species showed that the number of CesA genes ranged from the highest number in *V. planifolia* (15) to the lowest number in *P. aphrodite* (8). *D. officinale* had the highest number of each member in CslA (10), CslE (4), and CslG (7) subfamilies; while *C. ensifolium* had the highest number of each member in CslB (4) and CslH (3) subfamilies. CslH was found to be a unique subfamily in grasses (Farrokhi et al., 2006); however, it was identified in orchid species except for *D. huoshanense* and *G. elata*. The number of CslD subfamily members was highest (16) in *D. huoshanense* and lowest (6) in *G. elata*. Moreover, the CslF subfamily was lost in all orchid species. The distribution and divergence in the number of CesA/Csl subfamily members suggest that the CesA/Csl genes in the nine species with different life forms had undergone different evolutionary processes in Orchidaceae.

### Protein motif distribution and conserved domain analysis

**FIGURE 2:**
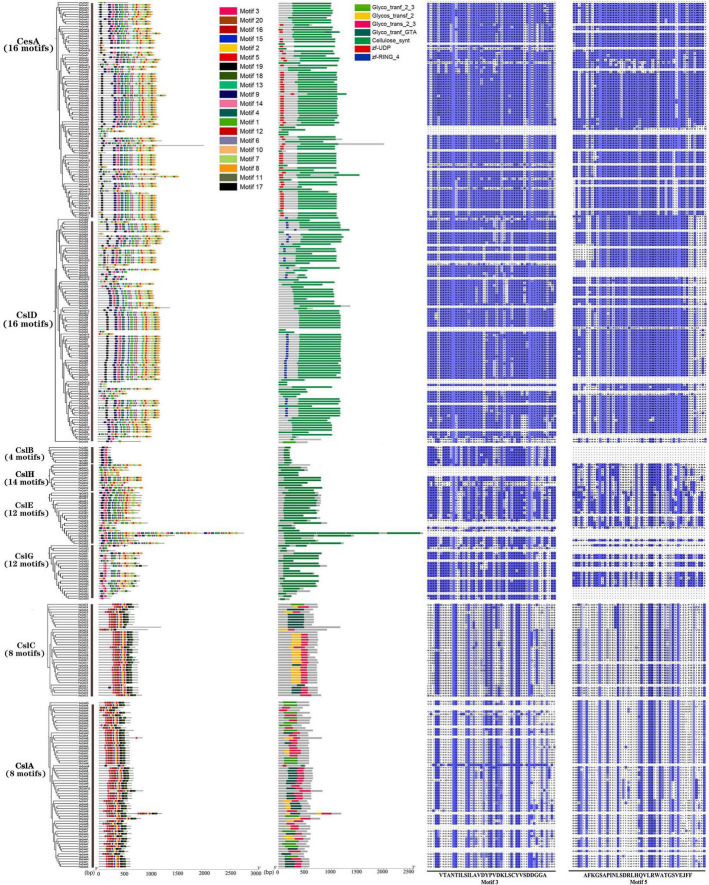
Protein motifs, conserved domains, and amino acid sequences of represented motifs (Motif 3 and Motif 5) corresponding eight CesA/Csl subfamilies in nine orchid species. Nine orchids have 20 types of motifs (Motif 1-Motif 20) and six types conserved domains: Cellulose_synt, Glyco_trans_2_3, Glyco_tranf_2_3, Glyco_tranf_2, Glyco_tranf_GTA_type, and zf_UDP.

Totally, 20 motifs and seven domains were identified from the orchid CesA/Csl superfamily (Figure 2, Supplementary Figure 1, and Supplementary Table 4). A comparison of the motifs and conserved domains of orchid proteins revealed that the CesA/Csl genes were conserved within different subfamilies but varied among subfamilies. Among all subfamilies, the types of motifs in CesA and CslD were exactly the same, both of which contained motif 1∼15 and motif 17 with the highest number (16) among all the subfamilies. CesA/CslD both contain common conserved domains Cellulose synt, but with unique domain zf-UDP in CesA and unique domain zf-RING_4 in CslD, respectively. Moreover, there were the same number (8) and types of protein motifs in the CslA and CslC subfamily (Supplementary Table 4). Both CslA and CslC uniquely contained motifs 16, 18, 19, and 20, but lacked 12 motifs (1,4,6,7,8,9,10,11,12,13,14,17), which might lead to the major sequence structural differences of CslA/CslC from other subfamilies (Supplementary Table 4). The conserved domains of both CslA and CslC were Glyco_trans_2_3, Glycos_transf_2, Glyco_tranf_GTA_type, and Glyco_tranf_2_3, all of which were conserved domains of Csl (Figure 2). In the CslB subfamily, there was the lowest number of motifs (4) with Cellulose_syn as a conserved domain. The motif distributions of the CslE, CslG, and CslH subfamilies were varied but with a single conserved Cellulose_syn domain. Overall, the CesA and CslD/E/G/H subfamilies that constituted 69.1% (241/349) of CesA/Csl genes were highly conserved, with 12–17 motifs and conserved Cellulose_synt domain. The CslA and CslC subfamilies accounted for 28.9% (101/349) of the CesA/Csl genes, and contained eight common motifs with four conserved domains. By multiple sequence alignments of the common motifs (motif 3 and motif 5) among subfamilies, it showed that the sequence of motif 3 in CslA/CslC had a larger variation compared with those of CesA/CslD, while the sequence of motif 5 in CslA/CslC had higher variations followed by that in CslE/CslG/CslH, while in CslD and CesA, motif 5 has the lowest variation (Figure 2 and Supplementary Figure 1).

### Selection pressure for CesA/Csl genes in orchids

**FIGURE 3:**
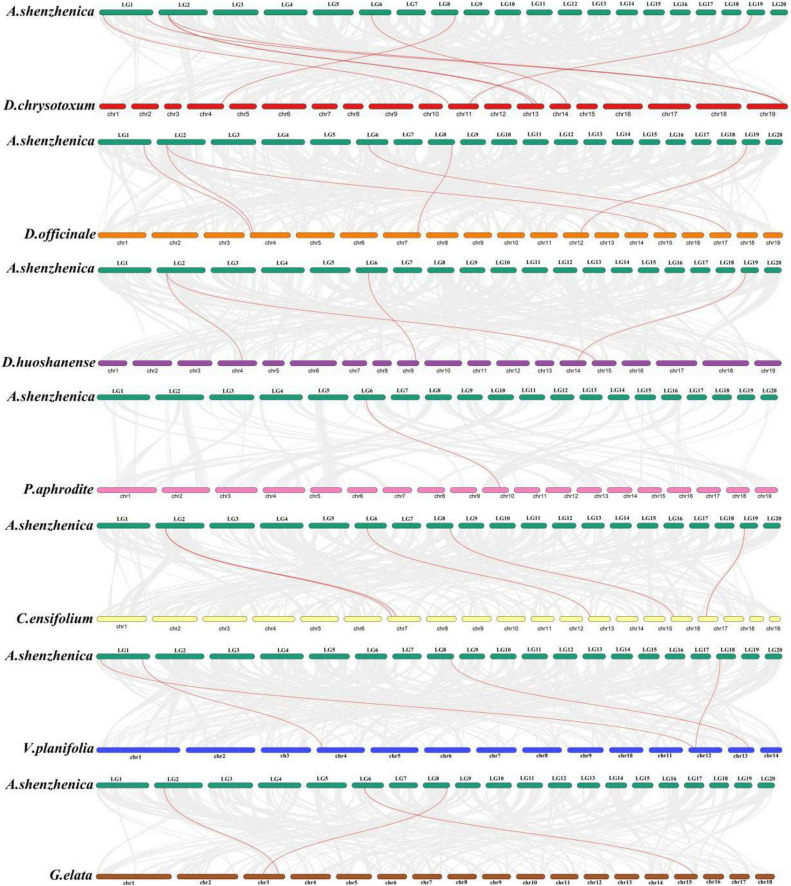
The collinearity diagram between *A. shenzhenica* and each of other eight orchid species. Red lines highlight the homologous gene pairs of CesA/Csl genes, and gray lines represent genome-wide collinear gene pairs.

The evolutionary selection pressure on CesA/Csl genes of the eight subfamilies among the nine orchid species was analyzed. Among all the gene-pairs comparisons, a total of 17 pairs of CesA/Csl genes with the *Ka*/*Ks* ratios > 1 (Figure 3). Five, five, one, two, and four gene pairs with *Ka*/*Ks* ratios > 1 were found in the CesA, CslD, CslA, CslE, and CslG subfamilies, respectively, indicating that members of these subfamilies underwent positive selection (Table 2). Among the members of the subfamilies subjected to positive selection, the CesA subfamily in *D. huoshanense* and *D. chrysotoxum* experienced the stronger positive selection, while the CslD subfamily in *D. officinale* had the highest number of positive selective genes. Additionally, the CslG subfamily in *D. officinale* and *D. chrysotoxum* experienced stronger positive selection. Moreover, the CslA subfamily in *V. planifolia* experienced positive selection; The positive selection pressure on the CslE subfamily in *P. aphrodite* was more than that in *C. ensifolium* and less than in *D. chrysotoxum*. The other four subfamilies, CslB, CslC, CslF, and CslH, had *Ka/Ks* ratios < 1, indicating that the members of these four subfamilies might have undergone purification selection. Overall, among the 17 CesA/Csl gene pairs that had undergone positive selection, 86% belonged to epiphytic orchids, 14% belonged to terrestrial orchids, while the saprophytic orchid *G. elata* did not undergo positive selection.

**TABLE 2:** Gene pairs with *Ka*/*Ks* > 1 in selection pressure analysis.

Subfamily	Seq_1	Seq_2	*Ka*	*Ks*	*Ka*/*Ks*
CesA	*DhCesA10*	*DcCesA3*	0.233	0.216	1.082
CesA	*DhCesA9*	*DcCesA3*	0.242	0.209	1.154
CesA	*DhCesA10*	*DcCesA4*	0.170	0.146	1.166
CesA	*PeCesA9*	*PeCesA5*	0.138	0.123	1.121
CesA	*DcCesA5*	*PeCesA9*	2.306	0.123	2.682
CslD	*VpCslD5*	*CeCslD5*	1.195	0.900	1.328
CslD	*VpCslD3*	*DhCslD14*	0.977	0.842	1.161
CslD	*DoCslD15*	*DoCslD14*	1.362	1.356	1.005
CslD	*DoCslD13*	*DoCslD3*	0.016	0.009	1.743
CslD	*DoCslD12*	*DoCslD3*	1.335	1.051	1.271
CslE	*PaCslE3*	*DcCslE1*	1.150	0.824	1.396
CslE	*PaCslE3*	*CeCslE2*	0.632	0.595	1.062
CslG	*DcCslG3*	*DcCslG1*	0.049	0.024	2.003
CslG	*DcCslG4*	*DcCslG1*	0.041	0.036	1.124
CslG	*DcCslG1*	*DoCslG6*	0.100	0.077	1.299
CslG	*DcCslG1*	*DoCslG7*	0.070	0.057	1.226
CslA	*VpCslA4*	*VpCslA3*	0.006	0.002	2.703

### Collinearity analysis of CesA/Csl gene family among orchids

Analysis of the collinearity relationship showed that the six *Ces/Csl* genes in the *A. shenzhenica* genome were collineated with eight homologous genes in the *D. chrysotoxum* genome (Figure 3). The *AsCslD1* gene in the *A. shenzhenica* genome collineated with three CslD genes in the *D. chrysotoxum* genome. Three *Ces/Csl* genes in the *A. shenzhenica* genome had four homologous genes in the *D. huoshanense* genome. Furthermore, the five *Ces/Csl* genes of *A. shenzhenica* had six homologous genes in the *D. officinale* genome. Among these, the CslD subfamily had expanded into two genes in *D. officinale* collineated with *the AsCslD1* gene in *A. shenzhenica*. The four *Ces/Csl* genes of *A. shenzhenica* had five homologous genes in the *C. ensifolium* genome, and the CslD subfamily genes in *C. ensifolium* had expanded into two genes collineated with *AsCslD1*. Moreover, three *Ces/Csl* genes in *A. shenzhenica* shared three homologous genes in the *G. elata* genome. *P. aphrodite* and *A. shenzhenica* had one pair of *Ces/Csl* homologous genes, while *V. planifolia* had four pairs of homologous genes with *AsCslC1* and *AsCslC3* contracted into one *VpCslC4* gene. Taking the CesA/Csl genes in *A. shenzhenica* as the references, among the corresponding homologous genes, two to three CslD subfamily genes had expanded in epiphytic *D. chrysotoxum*, *D. officinale*, *D. huoshanense*, and terrestrial *C. ensifolium*. However, CesA/Csl genes did not expand in epiphytic *P. aphrodite* and saprophytic *G. elata*, suggesting that the expansion and contraction of the CslD subfamily may be related to adaptive evolution to the local environment of each species during the evolution of different life forms in Orchidaceae.

### Genome-wide expression profiles of CesA/Csl genes in orchids

**FIGURE 4:**
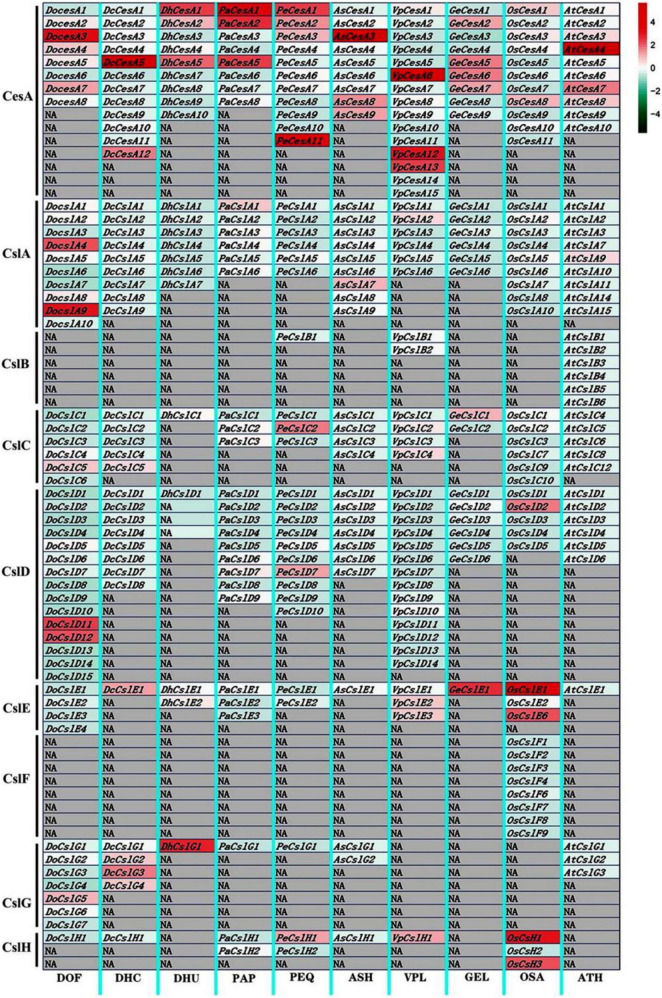
Heatmap of expression profiles of CesA/Csl genes in stem tissues of nine orchid species. The color bar indicates the expression level (normalized FPKM values) of cellulose synthase genes, with the red color indicating a high expression level, while the blue color represents a low expression level. The gray block indicates NA with no gene. The gene expression was normalized by each column respectively. DOF, *D. officinal*; DCH, *D. chrysotoxum*; DHU, *D. huoshanense*; PAP, *P. aphrodite*; PEQ, *P. equestris*; ASH, *A. shenzhenica*; VPL, *V. planifolia*; GEL, *G. elata*; OSA, *O. sativa*; ATH, *A. thaliana.*

We observed diversified expression patterns of the identified CesA/Csl genes in the stems tissues of eight orchid species, as well as that of *Arabidopsis* and *O. sativa*, using the FPKM expression matrix normalized by the column shown in the heat map (Figure 4 and Supplementary Table 2). The *CesA* genes of the CesA subfamily were strongly higher expressed among all the subfamilies in seven orchid species with epiphytic and terrestrial life forms. However, the expression level of CesA subfamily genes in saprophytic orchid *G. elata* exhibited weakly higher expression levels among nine subfamilies. The expression levels of genes in CslA (*DoCslA4* and *DoCslA9*) and CslD (*DoCslD11* and *DoCslD12*) subfamilies were higher among nine subfamilies in epiphytic *D. officinale*. The genes belonging to the CslC and CslG subfamilies did not show obvious upregulation expression patterns among subfamilies in each orchid species except *DhCslG1*. The *GeCslE1* in the CslE subfamily had the highest expression level among subfamilies in the saprophytic orchid *G. elata.* The genes in CslH subfamily were higher expressed among subfamilies in species *P. equestris* and *V. planifolia*. These results indicate that the CesA/Csl gene family has different biased expression patterns among subfamilies in orchid species, which implies that epiphytic, terrestrial, and saprophytic orchids had distinct adaptive expression regulation patterns related to cellulose synthase when adapting to different environments and stresses, of which CesA, CslA, and CslD subfamilies showed the most varied expression levels.

### Effects of abiotic stress on expression levels of CesA/Csl genes in orchids

**FIGURE 5:**
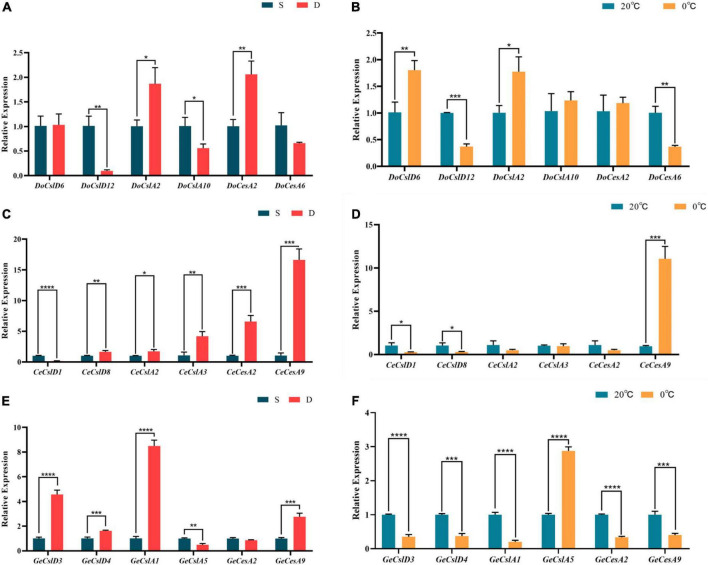
Histogram of the expression changes of selected genes of *D. officinale*, *C. ensifolium*, and *G. elata* under drought and low-temperature stress validated by qRT-PCR. **(A)** Gene expression changes of *D. officinale* under drought stress (S means normal watering, D means drought stress for 20 days, same below). **(B)** Gene expression changes of *D. officinale* under freezing stress (20°C, 0°C, and same below). **(C)** Gene expression changes of *C. ensifolium* under drought stress. **(D)** Gene expression changes of *C. ensifolium* under freezing stress. **(E)** Gene expression changes of *G. elata* under drought stress. **(F)** Gene expression changes of *G. elata* under freezing stress. *T*-tests were performed to determine whether expression changes were significant with ****P ≤ 0.0001; ***P ≤ 0.001; **P ≤ 0.01; *P ≤ 0.05.

To detect the changes in expression patterns of CesA/Csl genes in orchids with three different life forms under abiotic stress, three species (*D. officinale*, *C. ensifolium*, and *G. elata*) were selected as representatives of epiphytic, terrestrial, and saprophytic orchids, respectively, and the genes of CesA, CslA, and CslD subfamilies were selected to detect the gene expression changes under drought and freezing stresses in each orchid using real-time reverse transcription quantitative PCR (qRT-PCR; Figure 5 and Supplementary Table 5). The results showed that gene expression patterns in stems were either upregulated or downregulated for orchids with different life forms in response to drought and freezing stress (Figure 5). In the epiphytic orchid *D. officinale*, the expressions of *DoCslA2* and *DoCesA2* were upregulated under drought stress (*t*-test, *P* < 0.05), whereas the *DoCslD6* and *DoCslA2* were significantly increased under freezing stress (*t*-test, *P* < 0.001). The expression of genes *CeCslD8, CeCslA2, CeCslA3, CeCesA2* and *CeCesA9* in terrestrial orchid *C. ensifolium* was significantly increased under drought stress (*t*-test, *P* < 0.05), whereas *CeCesA9* strongly upregulated (*t*-test, *P* = 0.0003), and *CeCslD1* and *CeCslD8* slight downregulated (*t*-test, *P* < 0.05) under freezing stress. In the saprophytic orchid *G. elata*, the expression levels of *GeCslD3*, *GeCslD4*, *GeCslA1*, and *GeCesA9* were significantly upregulations under drought stress (*t*-test, *P* < 0.001), and expression levels of almost all genes were significantly decreased under freezing stress except *GeCslA5* (*t*-test, *P* < 0.05).

## Discussion

Orchidaceae is one of the few families of angiosperms that can successfully colonize terrestrial, epiphytic, or lithophytic ecological niches, such as land, trees, or rocks (Roberts and Dixon, 2008; Givnish et al., 2015, 2016). They are mainly divided into terrestrial, epiphytic, and saprophytic types based on their life forms under different environmental conditions (Cai et al., 2015; Zhang et al., 2016). Polysaccharides are the basic skeleton of plant cell walls and form the first line of defense that determines plant development and growth and helps plants resist adverse stress (Lampugnani et al., 2018; Zhang et al., 2020). Several studies showed that orchids especially those (e.g., *Dendrobium* orchids) with fleshy stems are rich in various types of active polysaccharides that are related to drought or cold stress adaptation (Xing et al., 2015; Jin et al., 2016; Zhang et al., 2016; Wan et al., 2018). The *CesA* and *Csl* genes that synthesize the β-1,4-linked glycan backbone of cellulose and hemicellulose polysaccharides encode enzymes related to cellulose and hemicellulose polysaccharides (Daras et al., 2021). Therefore, the systematic study of the *CesA* and *Csl* genes superfamily based on nine published orchid genomes and transcriptomes data has great significance for exploring the distribution and adaptive evolution of CesA/Csl genes among orchids with different life forms.

In this study, we identified 349 CesA/Csl genes in nine whole-genome sequenced species with different life forms (epiphytic, terrestrial, and saprophytic) in Orchidaceae. The evolutionary analysis of nine orchid species showed that CesA/Csl gene family members can be divided into eight subfamilies: CesA, and CslA/B/C/D/E/G/H (Figure 1), among which the CslD subfamily was the largest, followed by CesA, and the number of CslB subfamily members was the lowest, indicating that expansion of the CesA/Csl gene family in orchid species mainly occurred in the CslD subfamily, which is similar to the results of Lan et al. (2019). Although CslF and CslH only exist in grasses, the CslF subfamily was not identified in orchids in this study; however, we found that the CslH subfamily evolved in orchids, thereby adding new information to the evolution of CslH in monocots (Farrokhi et al., 2006). The identification of CesA/Csl gene family members in orchids showed that epiphytic orchid *D. officinale* contained the highest number of CesA/Csl genes (51), followed by terrestrial orchid *C. ensifolium* (48) and saprophytic orchid *G. elata* (24). Because *D. officinale* is an epiphyte and *G. elata* is a saprophyte, the different numbers of CesA/Csl genes might be related to the different life forms of orchid species. In addition, the distribution of each subfamily greatly differed in different orchid species. The CesA/Csl genes of *D. officinale*, *D. huoshanense*, and *V. planifolia* had expanded in the CslD subfamily. CslD is involved in the synthesis of xylan and galacturonic acid (Vogel, 2008). The synthesis of cellulose or mannan in the tip growth cells is closely related to the synthesis of metabolic components, such as polysaccharides (Park et al., 2011; Yang et al., 2020). In addition, both *D. officinale* and *D. huoshanense* are rich in various active polysaccharide ingredients (Jin et al., 2016). The expansion of CslD genes in these species might have played an important role in the synthesis of active polysaccharides for adapting to different environmental stress.

Analysis of protein motifs and conserved domains showed that the motifs and domains were strongly conserved within subfamilies in orchids. The CesA and CslD/E/G/H subfamilies, which have been found in orchid species were highly conserved, contained 12–16 motifs and Cellulose_synt conserved domains, and were involved in the synthesis of UDP and (1,4-β-D-glucosyl)n + 1 (Glaser, 1958). In addition, the CslA and CslC subfamilies contain eight motifs and four conserved domains (Glyco_trans2_3, Glyco_tranf_2_3, Glyco_tranf_2, and Glyco_tranf_GTA_type), all of which belong to glucosyltransferases involved in the biosynthesis of bacterial capsules (Swartley et al., 1998). Research has shown that the structures of genes in CesA and CslD, CslB, CslE, CslG, and CslH subfamilies are very similar (Daras et al., 2021). The members of CslA and CslC uniquely contained four motifs, but lacked 12 motifs, leading to the major structural differences with the other six CesA/Csl subfamilies, which are consistent with the results obtained in Daras et al. (2021).

Selection pressure analysis revealed that among the 17 CesA/Csl gene pairs that had undergone positive selection in orchids, 86%, 14%, and none belonged to epiphytic, terrestrial, and saprophytic orchids, respectively. Positive selection is one of the bases of adaption for the plant population evolution under local specific environmental conditions (Eyre-Walker, 2006). In our study, the evolutionary effects of positive selection on CesA/Csl were the strongest in epiphytic orchids, followed by the terrestrial orchids, and the weakest in the saprophytic orchids. The CesA/Csl genes of epiphytic orchids might have experienced stronger positive selection under drought and cold stress conditions in epiphytic environments than that in other environmental conditions (Roberts and Dixon, 2008; Givnish et al., 2015, 2016).

The inter-species collinearity analysis showed that the CslD subfamily in epiphytic *D. chrysotoxum*, *D. officinale*, and *D. huoshanense*, as well as terrestrial *C. ensifolium*, but not in terrestrial *P. aphrodite* and saprophytic *G. elata* had expanded into two to three genes collineated with *AsCslD1* gene in *A. shenzhenica*, suggesting that the expansion and contraction of the CslD subfamily might be related to adaptive evolution to environmental stress during the evolution of different life forms in Orchidaceae. Since CslD is located on the Golgi membrane and catalyzes the synthesis of 1,4-β-D-glucomannan from GDP glucose and GDP mannose (Huang et al., 2018; Yang et al., 2020). It is conserved in all terrestrial plants and is involved in various aspects of the plant life cycle, including polysaccharide synthesis (Zhang et al., 2016; Yu et al., 2018; Zhan, 2019), responses to environmental stimuli (Song et al., 2019), cell expansion and division (Yang et al., 2016; Peng et al., 2019), as well as plant development (Bernal et al., 2008; Luan et al., 2011; Yoo et al., 2012). Studies have shown that *DcCslDs* are differentially expressed in roots, stems, and leaves of *D. catenatum* using qRT-PCR (Xi et al., 2021). The drought-resistant recovery treatment was closely related to the expression level of *DcCslD5*, and low-temperature stress significantly affected the expression levels of *DcCslD1, DcCslD2a, DcCslD2b, DcCslD3a*, and *DcCslD5* (Xi et al., 2021). In our study, the *CslD* gene mainly expanded in epiphytic and partly in terrestrial orchids, but not in saprophytic orchids, which indicated that expansion of the *CslD* gene, which mainly synthesizes polysaccharides, might have promoted the adaptation of epiphytic orchids to extreme environments under positive selection.

Previous studies showed that the expression levels of CesA/Csl genes in the various tissues are differentiation which is also related to the abiotic stress in different species, such as Arabidopsis, rice, and orchids (Hamann et al., 2004; Kilian et al., 2007; Wang et al., 2010; Zhu et al., 2010; Lan et al., 2019; Gao et al., 2020; Xi et al., 2021). Our transcriptome analysis of eight orchid species (Figure 4) suggested that different CesA/Csl subfamilies with distinctly biased expression patterns in stems might be related to their adaptive ability to different environmental stresses. Further qRT-PCR results showed that the effects of both drought and cold stresses on the expression patterns of *CesA* genes were different in orchids with different life forms (Figure 5). In epiphytic orchid *D. officinale*, the CesA/Csl genes responded significantly to both drought and low-temperature stress. The CslA and CesA genes positively responded to drought stress, while the CslD and CslA genes positively responded to freezing stress (Figure 5). In terrestrial orchid *C. ensifolium*, the CslA and CesA genes were significantly upregulated under drought stress, while only the *CeCesA9* gene was significantly upregulated under freezing stress. The expression levels of CslD and CslA genes in saprophytic orchids *G. elata* were significantly upregulated under drought stress, but strongly downregulated under freezing stress, suggesting that saprophytic orchids were sensitive to both drought and cold stress by either up or down regulations of CesA/Csl genes. A previous study showed that the expression of *A. thaliana* CslA (*CslA7* and *CslA10*) and CslD (*CslD2* and *CslD3*) is induced under drought, cold, and osmotic stresses (Kilian et al., 2007; Zhu et al., 2010). The expression levels of *DcCslD5* are upregulated under drought stress in *D. catenatum*, while *DcCslD1*, *DcCslD2a*, *DcCslD2b*, *DcCslD3a*, and *DcCslD5* are influenced strongly by low temperature (Xi et al., 2021). Gao et al. (2020) showed that the promoter region of *CslA* genes contains cis-elements related to hormonal regulation, which play an important role in biosynthesis and accumulation of glucomannan that responds to stress in *D. catenatum*. Under different stress treatments, low temperatures induced the expression of *DcCslA5* and inhibited the expression of *DcCslA3* (Gao et al., 2020). Combining with previous studies as well as our studies of gene expressions and positive selection, we proposed that the CslA might be a key subfamily in orchids with different life forms in response to drought stress, while the CslD might be a key subfamily in epiphytic and saprophytic orchids to adapt to freezing stress.

## Conclusion

In this study, 349 CesA/Csl genes were identified in nine orchid species with different life forms (epiphytic, terrestrial, and saprophytic) in Orchidaceae. The CesA/Csl superfamily in orchids was divided into eight subfamilies: CesA and CslA/B/C/D/E/G/H with conserved motifs and domains. Totally, 17 pairs of CesA/Csl homologous genes underwent positive selection, of which 86%, 14%, and none belonged to the epiphytic, terrestrial, and saprophytic orchids, respectively. Epiphytic orchids experienced greater strength of positive selection, with expansion events mostly related to the CslD subfamily, which might have resulted in strong adaptability to stress in epiphytes. Epiphytic, terrestrial, and saprophytic orchids had different adaptive expression patterns. CslA might be a key subfamily in orchids with different life forms in response to drought stress, whereas the CslD subfamily might be a key subfamily in epiphytic and saprophytic orchids to adapt to freezing stress. The novel findings in the study will provide the basic resource for further research on the adaptive evolution of the CesA/Csl superfamily in angiosperms, as well as the orchid-specific functional genes related to life-history trait evolution.

## Data availability statement

The datasets presented in this study can be found in online repositories. The names of the repository/repositories and accession number(s) can be found in the article/Supplementary material.

## Authors contributions

XM conceived the project and designed the experiments. JW, JL, WL, BD, LL, and XL carried out the experiments. JW and XM analyzed the data and wrote the manuscript. QH and KL visualized the experimental results. XM, DQ, BH, and MF revised the manuscript. All authors read and approved the final manuscript.

## Footnotes

1
https://db.cngb.org/cnsa/

2
https://www.ncbi.nlm.nih.gov/

3
http://rice.uga.edu

4
https://www.arabidopsis.org/

5
https://www.ncbi.nlm.nih.gov/sra/

6
http://pfam.xfam.org

7
https://meme-suite.org/meme/tools/meme

8
https://www.ncbi.nlm.nih.gov/cdd/

9
https://github.com/FelixKrueger/TrimGalore
