# Supplementary material for: Genome-wide identification and adaptive evolution of CesA/Csl superfamily among species with different life forms in Orchidaceae
Source: Front Plant Sci. 2022 Sep 29;13:994679. doi: 10.3389/fpls.2022.994679 (PMC9559377; doi:10.3389/fpls.2022.994679)
Supplement: Supplementary file 1 [file Data_Sheet_1.pdf]

# Supplementary materials

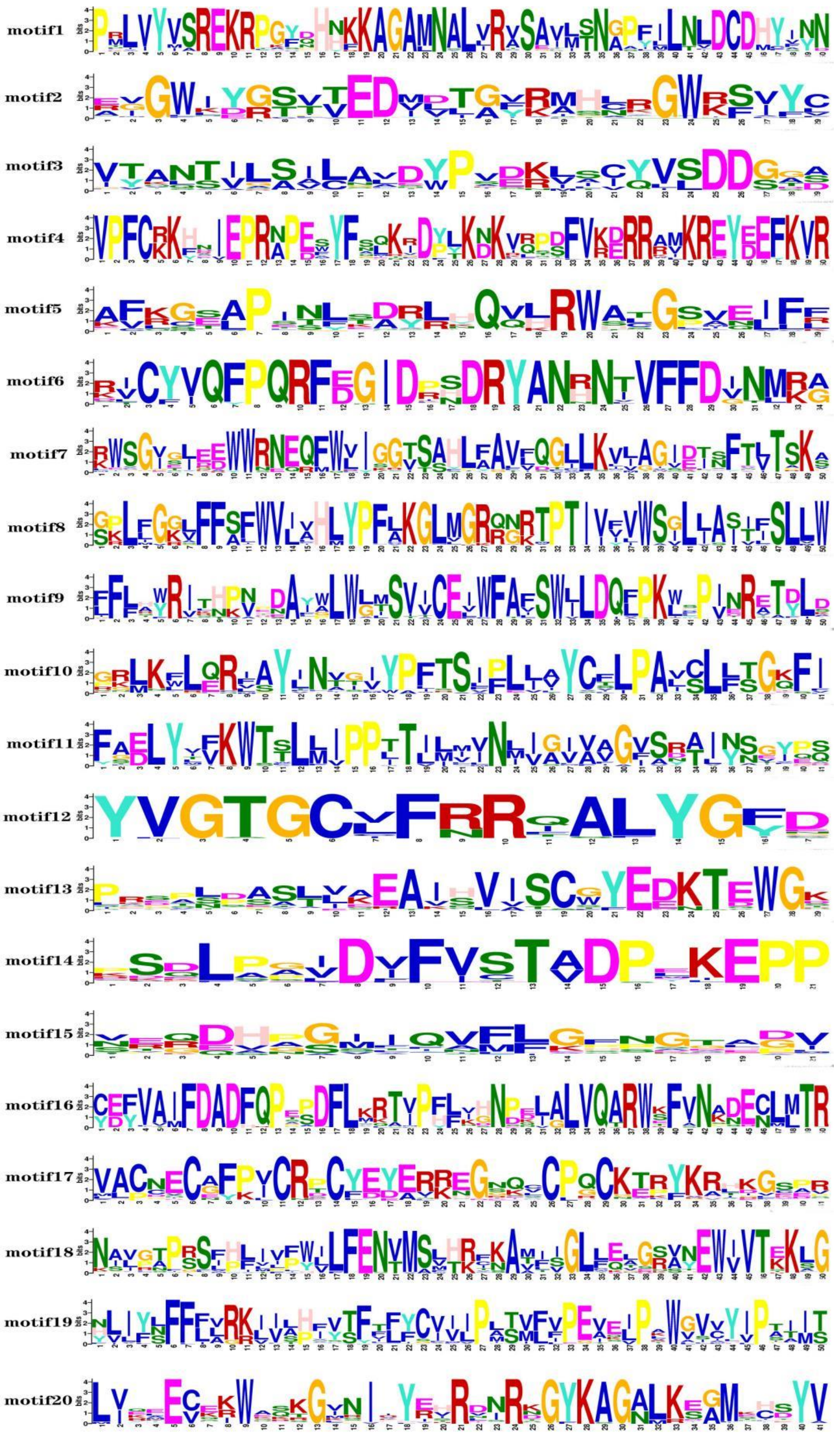


**Supplementary Figure 1. Logo structures from Motif 1 to Motif 20.**

**Supplementary Table 1. Genome and protein sequence sources of 11 species used in this study.**

| Species | life style | Genome data link |
|---|---|---|
| *D.officinale* | Epiphyte | https://www.ncbi.nlm.nih.gov/genome/?term=JACXSL000000000 |
| *D.huoshanense* | Epiphyte | https://ftp.cngb.org/pub/CNSA/data3/CNP0000830/CNS0251991/CNA0014590/ |
| *D.chrysotoxum* | Epiphyte | https://ftp.ncbi.nlm.nih.gov/genomes/all/GCA/019/925/795/GCA_019925795.1_ASM1992579v1/ |
| *P.aphrodite* | Epiphyte | http://orchidstra2.abrc.sinica.edu.tw/orchidstra2/padownload.php |
| *P.equestris* | Epiphyte | https://ftp.cngb.org/pub/Assembly/GCF/001/263/595/GCF_001263595.1_ASM126359v1/ |
| *C.ensifolium* | Terrestrial | https://ngdc.cncb.ac.cn/search/?dbId=gwh&q=GWHBCII00000000&page=1 |
| *V.planifolia* | Terrestrial | https://ftp.ncbi.nlm.nih.gov/genomes/all/GCA/016/413/895/GCA_016413895.1_Elo_Vpla-A_principal_1.0/ |
| *A.shenzhenica* | Terrestrial | https://ftp.ncbi.nlm.nih.gov/genomes/all/GCA/002/786/265/GCA_002786265.1_ASM278626v1/ |
| *G.elata* | Saprophytic | https://ngdc.cncb.ac.cn/search/?dbId=gwh&q=Gastrodia+elata+ |
| *A.thaliana* | Terrestrial | ftp://ftp.ensemblgenomes.org/pub/plants/release-41/gff3/ |
| *O.sativa* | Terrestrial | http://rice.uga.edu/pub/data/Eukaryotic_Projects/o_sativa/annotation_dbs/pseudomolecules/version_7.0/all.dir/ |

**Supplementary Table 2. Transcriptome data sources of 10 species used in study**

| Species | Tissue | Age | RNA-seq raw data link |
|---|---|---|---|
| *D.officinale* | stem | 2 years | https://www.ncbi.nlm.nih.gov/Traces/study/?acc=SRP344145&o=acc_s%3Aa |
| *D.huoshanense* | stem | 2 years | https://www.ncbi.nlm.nih.gov/Traces/study/?acc=SRP291861&o=acc_s%3Aa |
| *D.chrysotoxum* | stem | 4 years | https://www.ncbi.nlm.nih.gov/Traces/study/?acc=SRP287345&o=acc_s%3Aa |
| *P.aphrodite* | stalk | 1 year | https://www.ncbi.nlm.nih.gov/Traces/study/?acc=SRP090595&o=acc_s%3Aa |
| *P.equestris* | stem | wild mature | https://www.ncbi.nlm.nih.gov/Traces/study/?acc=SRP074449&o=acc_s%3Aa |
| *V.planifolia* | stem | 1 year | https://www.ncbi.nlm.nih.gov/Traces/study/?acc=SRP043630&o=acc_s%3Aa |
| *A.shenzhenica* | stem | 3 years | https://www.ncbi.nlm.nih.gov/Traces/study/?acc=SRP109877&o=acc_s%3Aa |
| *G.elata* | tuber | 2 years | https://www.ncbi.nlm.nih.gov/Traces/study/?acc=SRP108465&o=acc_s%3Aa |
| *A.thaliana* | stem | 4 weeks | https://www.ncbi.nlm.nih.gov/Traces/study/?acc=SRP150048&o=acc_s%3Aa |
| *O.sativa* | stem | freshly harvest | https://www.ncbi.nlm.nih.gov/Traces/study/?acc=SRP163921&o=acc_s%3Aa |

**Supplementary Table 3. lists of CesA/Csl gene family member identified and gene renaming.**

| NO. | Subfamily | geneID | Gene name | NO. | Subfamily | GeneID | Gene name |
|---|---|---|---|---|---|---|---|
| 1 | CesA | AXF42_Ash002151 | *AsCesA1* | 218 | CslC | AT4G07960.1 | *AtCslC12* |
| 2 | CesA | AXF42_Ash004400 | *AsCesA2* | 219 | CslC | AT4G31590.1 | *AtCslC5* |
| 3 | CesA | AXF42_Ash010549 | *AsCesA3* | 220 | CslC | JL001739 | *CeCslC1* |
| 4 | CesA | AXF42_Ash011326 | *AsCesA4* | 221 | CslC | JL009311 | *CeCslC2* |
| 5 | CesA | AXF42_Ash011671 | *AsCesA5* | 222 | CslC | JL012459 | *CeCslC3* |
| 6 | CesA | AXF42_Ash014045 | *AsCesA6* | 223 | CslC | JL019560 | *CeCslC4* |
| 7 | CesA | AXF42_Ash016818 | *AsCesA7* | 224 | CslC | JL023838 | *CeCslC5* |
| 8 | CesA | AXF42_Ash018579 | *AsCesA8* | 225 | CslC | IEQ34_002245 | *DcCslC1* |
| 9 | CesA | AXF42_Ash020427 | *AsCesA9* | 226 | CslC | IEQ34_012217 | *DcCslC2* |
| 10 | CesA | AT2G21770.1 | *AtCesA9* | 227 | CslC | IEQ34_012267 | *DcCslC3* |
| 11 | CesA | AT2G25540.1 | *AtCesA10* | 228 | CslC | IEQ34_013796 | *DcCslC4* |
| 12 | CesA | AT4G18780.1 | *AtCesA8* | 229 | CslC | IEQ34_022873 | *DcCslC5* |
| 13 | CesA | AT4G32410.1 | *AtCesA1* | 230 | CslC | Dhu000017871 | *DhCslC1* |
| 14 | CesA | AT4G39350.1 | *AtCesA2* | 231 | CslC | Dhu000021363 | *DhCslC2* |
| 15 | CesA | AT5G05170.1 | *AtCesA3* | 232 | CslC | Dhu000025164 | *DhCslC3* |
| 16 | CesA | AT5G09870.1 | *AtCesA5* | 233 | CslC | Dhu000025835 | *DhCslC4* |
| 17 | CesA | AT5G17420.1 | *AtCesA7* | 234 | CslC | dof06625 | *DoCslC1* |
| 18 | CesA | AT5G44030.1 | *AtCesA4* | 235 | CslC | dof06636 | *DoCslC2* |
| 19 | CesA | AT5G64740.1 | *AtCesA6* | 236 | CslC | dof18577 | *DoCslC3* |
| 20 | CesA | JL003376 | *CeCesA1* | 237 | CslC | dof19470 | *DoCslC4* |
| 21 | CesA | JL012073 | *CeCesA2* | 238 | CslC | dof20547 | *DoCslC5* |
| 22 | CesA | JL014002 | *CeCesA3* | 239 | CslC | dof26813 | *DoCslC6* |
| 23 | CesA | JL016086 | *CeCesA4* | 240 | CslC | GelC08G00230 | *GeCslC1* |
| 24 | CesA | JL017919 | *CeCesA5* | 241 | CslC | GelC18G00282 | *GeCslC2* |
| 25 | CesA | JL022406 | *CeCesA6* | 242 | CslC | LOC_Os01g56130.1 | *OsCslC1* |
| 26 | CesA | JL023672 | *CeCesA7* | 243 | CslC | LOC_Os03g56060.1 | *OsCslC9* |
| 27 | CesA | JL023776 | *CeCesA8* | 244 | CslC | LOC_Os05g43530.1 | *OsCslC7* |
| 28 | CesA | JL024122 | *CeCesA9* | 245 | CslC | LOC_Os07g03260.1 | *OsCslC10* |
| 29 | CesA | JL024520 | *CeCesA10* | 246 | CslC | LOC_Os08g15420.1 | *OsCslC3* |
| 30 | CesA | IEQ34_001218 | *DcCesA1* | 247 | CslC | LOC_Os09g25900.1 | *OsCslC2* |
| 31 | CesA | IEQ34_003409 | *DcCesA2* | 248 | CslC | PAXXG018280 | *PaCslC1* |
| 32 | CesA | IEQ34_008033 | *DcCesA3* | 249 | CslC | PAXXG167820 | *PaCslC2* |
| 33 | CesA | IEQ34_008239 | *DcCesA4* | 250 | CslC | PAXXG248380 | *PaCslC3* |
| 34 | CesA | IEQ34_008597 | *DcCesA5* | 251 | CslC | LOC110023409 | *PeCslC1* |
| 35 | CesA | IEQ34_009851 | *DcCesA6* | 252 | CslC | LOC110026863 | *PeCslC2* |
| 36 | CesA | IEQ34_010462 | *DcCesA7* | 253 | CslC | LOC110035933 | *PeCslC3* |
| 37 | CesA | IEQ34_011495 | *DcCesA8* | 254 | CslC | HPP92_006961 | *VpCslC1* |
| 38 | CesA | IEQ34_013110 | *DcCesA9* | 255 | CslC | HPP92_012265 | *VpCslC2* |
| 39 | CesA | IEQ34_013802 | *DcCesA10* | 256 | CslC | HPP92_015854 | *VpCslC3* |
| 40 | CesA | IEQ34_013803 | *DcCesA11* | 257 | CslC | HPP92_019574 | *VpCslC4* |
| 41 | CesA | IEQ34_019272 | *DcCesA12* | 258 | CslD | AXF42_Ash000941 | *AsCslD1* |
| 42 | CesA | Dhu000003787 | *DhCesA1* | 259 | CslD | AXF42_Ash007779 | *AsCslD2* |
| 43 | CesA | Dhu000008922 | *DhCesA2* | 260 | CslD | AXF42_Ash009314 | *AsCslD3* |

| | | | | | | | |
|---|---|---|---|---|---|---|---|
| 44 | CesA | Dhu000010663 | *DhCesA3* | 261 | CslD | AXF42_Ash009972 | *AsCslD4* |
| 45 | CesA | Dhu000015627 | *DhCesA4* | 262 | CslD | AXF42_Ash012026 | *AsCslD5* |
| 46 | CesA | Dhu000021441 | *DhCesA5* | 263 | CslD | AXF42_Ash019363 | *AsCslD6* |
| 47 | CesA | Dhu000024765 | *DhCesA6* | 264 | CslD | AXF42_Ash019601 | *AsCslD7* |
| 48 | CesA | Dhu000025130 | *DhCesA7* | 265 | CslD | AT1G02730.1 | *AtCslD5* |
| 49 | CesA | Dhu000025372 | *DhCesA8* | 266 | CslD | AT1G32180.1 | *AtCslD6* |
| 50 | CesA | Dhu000025586 | *DhCesA9* | 267 | CslD | AT2G33100.1 | *AtCslD1* |
| 51 | CesA | Dhu000025590 | *DhCesA10* | 268 | CslD | AT3G03050.1 | *AtCslD3* |
| 52 | CesA | dof05000 | *DoCesA1* | 269 | CslD | AT4G38190.1 | *AtCslD4* |
| 53 | CesA | dof12654 | *DoCesA2* | 270 | CslD | AT5G16910.1 | *AtCslD2* |
| 54 | CesA | dof12667 | *DoCesA3* | 271 | CslD | JL004961 | *CeCslD1* |
| 55 | CesA | dof13827 | *DoCesA4* | 272 | CslD | JL005906 | *CeCslD2* |
| 56 | CesA | dof17865 | *DoCesA5* | 273 | CslD | JL005963 | *CeCslD3* |
| 57 | CesA | dof20692 | *DoCesA6* | 274 | CslD | JL007154 | *CeCslD4* |
| 58 | CesA | dof21280 | *DoCesA7* | 275 | CslD | JL007155 | *CeCslD5* |
| 59 | CesA | dof24252 | *DoCesA8* | 276 | CslD | JL009535 | *CeCslD6* |
| 60 | CesA | GelC02G00775 | *GeCesA1* | 277 | CslD | JL011091 | *CeCslD7* |
| 61 | CesA | GelC03G00800 | *GeCesA2* | 278 | CslD | JL013177 | *CeCslD8* |
| 62 | CesA | GelC03G00803 | *GeCesA3* | 279 | CslD | JL025396 | *CeCslD9* |
| 63 | CesA | GelC05G00571 | *GeCesA4* | 280 | CslD | IEQ34_000395 | *DcCslD1* |
| 64 | CesA | GelC08G00414 | *GeCesA5* | 281 | CslD | IEQ34_000854 | *DcCslD2* |
| 65 | CesA | GelC08G00833 | *GeCesA6* | 282 | CslD | IEQ34_011871 | *DcCslD3* |
| 66 | CesA | GelC08G00834 | *GeCesA7* | 283 | CslD | IEQ34_014165 | *DcCslD4* |
| 67 | CesA | GelC16G00614 | *GeCesA8* | 284 | CslD | IEQ34_014785 | *DcCslD5* |
| 68 | CesA | GelC18G00104 | *GeCesA9* | 285 | CslD | IEQ34_017247 | *DcCslD6* |
| 69 | CesA | LOC_Os01g54620.1 | *OsCesA4* | 286 | CslD | IEQ34_022283 | *DcCslD7* |
| 70 | CesA | LOC_Os03g59340.1 | *OsCesA2* | 287 | CslD | IEQ34_022675 | *DcCslD8* |
| 71 | CesA | LOC_Os03g62090.1 | *OsCesA5* | 288 | CslD | Dhu000000307 | *DhCslD1* |
| 72 | CesA | LOC_Os05g08370.1 | *OsCesA1* | 289 | CslD | Dhu000001763 | *DhCslD2* |
| 73 | CesA | LOC_Os06g39970.1 | *OsCesA11* | 290 | CslD | Dhu000003130 | *DhCslD3* |
| 74 | CesA | LOC_Os07g10770.1 | *OsCesA8* | 291 | CslD | Dhu000003132 | *DhCslD4* |
| 75 | CesA | LOC_Os07g14850.1 | *OsCesA6* | 292 | CslD | Dhu000004519 | *DhCslD5* |
| 76 | CesA | LOC_Os07g24190.1 | *OsCesA3* | 293 | CslD | Dhu000006138 | *DhCslD6* |
| 77 | CesA | LOC_Os09g25490.1 | *OsCesA9* | 294 | CslD | Dhu000008804 | *DhCslD7* |
| 78 | CesA | LOC_Os10g32980.1 | *OsCesA7* | 295 | CslD | Dhu000009340 | *DhCslD8* |
| 79 | CesA | LOC_Os12g29300.1 | *OsCesA10* | 296 | CslD | Dhu000011652 | *DhCslD9* |
| 80 | CesA | PAXXG022840 | *PaCesA1* | 297 | CslD | Dhu000015183 | *DhCslD10* |
| 81 | CesA | PAXXG024350 | *PaCesA2* | 298 | CslD | Dhu000015190 | *DhCslD11* |
| 82 | CesA | PAXXG038540 | *PaCesA3* | 299 | CslD | Dhu000018338 | *DhCslD12* |
| 83 | CesA | PAXXG058770 | *PaCesA4* | 300 | CslD | Dhu000020046 | *DhCslD13* |
| 84 | CesA | PAXXG223370 | *PaCesA5* | 301 | CslD | Dhu000021195 | *DhCslD14* |
| 85 | CesA | PAXXG233620 | *PaCesA6* | 302 | CslD | Dhu000021197 | *DhCslD15* |
| 86 | CesA | PAXXG240880 | *PaCesA7* | 303 | CslD | Dhu000027930 | *DhCslD16* |
| 87 | CesA | PAXXG260600 | *PaCesA8* | 304 | CslD | dof06816 | *DoCslD1* |
| 88 | CesA | LOC110018509 | *PeCesA1* | 305 | CslD | dof07142 | *DoCslD2* |
| 89 | CesA | LOC110018542 | *PeCesA2* | 306 | CslD | dof07176 | *DoCslD3* |
| 90 | CesA | LOC110021714 | *PeCesA3* | 307 | CslD | dof09423 | *DoCslD4* |

| | | | | | | | |
|---|---|---|---|---|---|---|---|
| 91 | CesA | LOC110022035 | *PeCesA4* | 308 | CslD | dof10592 | *DoCslD5* |
| 92 | CesA | LOC110023735 | *PeCesA5* | 309 | CslD | dof14185 | *DoCslD6* |
| 93 | CesA | LOC110025906 | *PeCesA6* | 310 | CslD | dof18176 | *DoCslD7* |
| 94 | CesA | LOC110032300 | *PeCesA7* | 311 | CslD | dof20722 | *DoCslD8* |
| 95 | CesA | LOC110033333 | *PeCesA8* | 312 | CslD | dof22452 | *DoCslD9* |
| 96 | CesA | LOC110034229 | *PeCesA9* | 313 | CslD | dof22475 | *DoCslD10* |
| 97 | CesA | LOC110034708 | *PeCesA10* | 314 | CslD | dof22743 | *DoCslD11* |
| 98 | CesA | LOC110035201 | *PeCesA11* | 315 | CslD | dof22744 | *DoCslD12* |
| 99 | CesA | HPP92_006832 | *VpCesA1* | 316 | CslD | dof28243 | *DoCslD13* |
| 100 | CesA | HPP92_007787 | *VpCesA2* | 317 | CslD | dof29098 | *DoCslD14* |
| 101 | CesA | HPP92_012648 | *VpCesA3* | 318 | CslD | dof29099 | *DoCslD15* |
| 102 | CesA | HPP92_016155 | *VpCesA4* | 319 | CslD | GelC03G01042 | *GeCslD1* |
| 103 | CesA | HPP92_016156 | *VpCesA5* | 320 | CslD | GelC03G01285 | *GeCslD2* |
| 104 | CesA | HPP92_017796 | *VpCesA6* | 321 | CslD | GelC06G00726 | *GeCslD3* |
| 105 | CesA | HPP92_018340 | *VpCesA7* | 322 | CslD | GelC07G00393 | *GeCslD4* |
| 106 | CesA | HPP92_018867 | *VpCesA8* | 323 | CslD | GelC09G00325 | *GeCslD5* |
| 107 | CesA | HPP92_018873 | *VpCesA9* | 324 | CslD | GelC16G00224 | *GeCslD6* |
| 108 | CesA | HPP92_019937 | *VpCesA10* | 325 | CslD | LOC_Os06g02180.1 | *OsCslD2* |
| 109 | CesA | HPP92_019938 | *VpCesA11* | 326 | CslD | LOC_Os06g22980.1 | *OsCslD5* |
| 110 | CesA | HPP92_021811 | *VpCesA12* | 327 | CslD | LOC_Os08g25710.1 | *OsCslD3* |
| 111 | CesA | HPP92_024874 | *VpCesA13* | 328 | CslD | LOC_Os10g42750.1 | *OsCslD1* |
| 112 | CesA | HPP92_027358 | *VpCesA14* | 329 | CslD | LOC_Os12g36890.1 | *OsCslD4* |
| 113 | CesA | HPP92_027359 | *VpCesA15* | 330 | CslD | PAXXG000590 | *PaCslD1* |
| 114 | CslA | AXF42_Ash003303 | *AsCslA1* | 331 | CslD | PAXXG003790 | *PaCslD2* |
| 115 | CslA | AXF42_Ash010752 | *AsCslA2* | 332 | CslD | PAXXG023130 | *PaCslD3* |
| 116 | CslA | AXF42_Ash011774 | *AsCslA3* | 333 | CslD | PAXXG120890 | *PaCslD4* |
| 117 | CslA | AXF42_Ash011839 | *AsCslA4* | 334 | CslD | PAXXG150250 | *PaCslD5* |
| 118 | CslA | AXF42_Ash014756 | *AsCslA5* | 335 | CslD | PAXXG156700 | *PaCslD6* |
| 119 | CslA | AXF42_Ash016464 | *AsCslA6* | 336 | CslD | PAXXG188550 | *PaCslD7* |
| 120 | CslA | AXF42_Ash016465 | *AsCslA7* | 337 | CslD | PAXXG228240 | *PaCslD8* |
| 121 | CslA | AXF42_Ash016466 | *AsCslA8* | 338 | CslD | PAXXG228570 | *PaCslD9* |
| 122 | CslA | AXF42_Ash017592 | *AsCslA9* | 339 | CslD | LOC110020746 | *PeCslD1* |
| 123 | CslA | AT1G23480.1 | *AtCslA3* | 340 | CslD | LOC110021713 | *PeCslD2* |
| 124 | CslA | AT1G24070.1 | *AtCslA10* | 341 | CslD | LOC110022849 | *PeCslD3* |
| 125 | CslA | AT2G35650.1 | *AtCslA7* | 342 | CslD | LOC110023622 | *PeCslD4* |
| 126 | CslA | AT3G56000.1 | *AtCslA14* | 343 | CslD | LOC110023929 | *PeCslD5* |
| 127 | CslA | AT4G13410.1 | *AtCslA15* | 344 | CslD | LOC110024006 | *PeCslD6* |
| 128 | CslA | AT4G16590.1 | *AtCslA1* | 345 | CslD | LOC110026391 | *PeCslD7* |
| 129 | CslA | AT5G03760.1 | *AtCslA9* | 346 | CslD | LOC110031409 | *PeCslD8* |
| 130 | CslA | AT5G16190.1 | *AtCslA11* | 347 | CslD | LOC110037910 | *PeCslD9* |
| 131 | CslA | AT5G22740.1 | *AtCslA2* | 348 | CslD | LOC110038752 | *PeCslD10* |
| 132 | CslA | JL002003 | *CeCslA1* | 349 | CslD | HPP92_002974 | *VpCslD1* |
| 133 | CslA | JL002005 | *CeCslA2* | 350 | CslD | HPP92_002984 | *VpCslD2* |
| 134 | CslA | JL011124 | *CeCslA3* | 351 | CslD | HPP92_002985 | *VpCslD3* |
| 135 | CslA | JL011525 | *CeCslA4* | 352 | CslD | HPP92_002986 | *VpCslD4* |
| 136 | CslA | JL013721 | *CeCslA5* | 353 | CslD | HPP92_002992 | *VpCslD5* |
| 137 | CslA | JL015007 | *CeCslA6* | 354 | CslD | HPP92_002993 | *VpCslD6* |

| | | | | | | | |
|---|---|---|---|---|---|---|---|
| 138 | CslA | JL020340 | *CeCslA7* | 355 | CslD | HPP92_005275 | *VpCslD7* |
| 139 | CslA | IEQ34_010633 | *DcCslA1* | 356 | CslD | HPP92_005276 | *VpCslD8* |
| 140 | CslA | IEQ34_011484 | *DcCslA2* | 357 | CslD | HPP92_014698 | *VpCslD9* |
| 141 | CslA | IEQ34_015323 | *DcCslA3* | 358 | CslD | HPP92_015208 | *VpCslD10* |
| 142 | CslA | IEQ34_015957 | *DcCslA4* | 359 | CslD | HPP92_016061 | *VpCslD11* |
| 143 | CslA | IEQ34_018066 | *DcCslA5* | 360 | CslD | HPP92_016083 | *VpCslD12* |
| 144 | CslA | IEQ34_018401 | *DcCslA6* | 361 | CslD | HPP92_022063 | *VpCslD13* |
| 145 | CslA | IEQ34_019977 | *DcCslA7* | 362 | CslD | HPP92_022064 | *VpCslD14* |
| 146 | CslA | IEQ34_021411 | *DcCslA8* | 363 | CslE | AXF42_Ash010839 | *AsCslE1* |
| 147 | CslA | Dhu000001868 | *DhCslA1* | 364 | CslE | AT1G55850.1 | *AtCslE1* |
| 148 | CslA | Dhu000009072 | *DhCslA2* | 365 | CslE | JL017554 | *CeCslE1* |
| 149 | CslA | Dhu000015542 | *DhCslA3* | 366 | CslE | JL017555 | *CeCslE2* |
| 150 | CslA | Dhu000015824 | *DhCslA4* | 367 | CslE | JL028771 | *CeCslE3* |
| 151 | CslA | Dhu000015826 | *DhCslA5* | 368 | CslE | JL028933 | *CeCslE4* |
| 152 | CslA | Dhu000020499 | *DhCslA6* | 369 | CslE | IEQ34_008753 | *DcCslE1* |
| 153 | CslA | Dhu000024845 | *DhCslA7* | 370 | CslE | Dhu000026047 | *DhCslE1* |
| 154 | CslA | dof00215 | *DoCslA1* | 371 | CslE | Dhu000026049 | *DhCslE2* |
| 155 | CslA | dof08220 | *DoCslA2* | 372 | CslE | dof06089 | *DoCslE1* |
| 156 | CslA | dof09755 | *DoCslA3* | 373 | CslE | dof06091 | *DoCslE2* |
| 157 | CslA | dof09756 | *DoCslA4* | 374 | CslE | dof06092 | *DoCslE3* |
| 158 | CslA | dof09758 | *DoCslA5* | 375 | CslE | dof06093 | *DoCslE4* |
| 159 | CslA | dof17125 | *DoCslA6* | 376 | CslE | GelC04G01180 | *GeCslE1* |
| 160 | CslA | dof23172 | *DoCslA7* | 377 | CslE | LOC_Os02g49332.1 | *OsCslE2* |
| 161 | CslA | dof25145 | *DoCslA8* | 378 | CslE | LOC_Os09g30120.1 | *OsCslE1* |
| 162 | CslA | dof25146 | *DoCslA9* | 379 | CslE | LOC_Os09g30130.1 | *OsCslE6* |
| 163 | CslA | dof25148 | *DoCslA10* | 380 | CslE | PAXXG089370 | *PaCslE1* |
| 164 | CslA | GelC02G00958 | *GeCslA1* | 381 | CslE | PAXXG089380 | *PaCslE2* |
| 165 | CslA | GelC05G01348 | *GeCslA2* | 382 | CslE | PAXXG089400 | *PaCslE3* |
| 166 | CslA | GelC10G01017 | *GeCslA3* | 383 | CslE | LOC110027276 | *PeCslE1* |
| 167 | CslA | GelC11G00994 | *GeCslA4* | 384 | CslE | LOC110027324 | *PeCslE2* |
| 168 | CslA | GelC11G00995 | *GeCslA5* | 385 | CslE | HPP92_007502 | *VpCslE1* |
| 169 | CslA | GelC15G00682 | *GeCslA6* | 386 | CslE | HPP92_022824 | *VpCslE2* |
| 170 | CslA | LOC_Os02g09930.1 | *OsCslA1* | 387 | CslE | HPP92_022825 | *VpCslE3* |
| 171 | CslA | LOC_Os02g51060.1 | *OsCslA6* | 388 | CslF | LOC_Os07g36610.1 | *OsCslF9* |
| 172 | CslA | LOC_Os03g07350.1 | *OsCslA4* | 389 | CslF | LOC_Os07g36630.1 | *OsCslF8* |
| 173 | CslA | LOC_Os03g26044.1 | *OsCslA5* | 390 | CslF | LOC_Os07g36690.1 | *OsCslF2* |
| 174 | CslA | LOC_Os06g12460.1 | *OsCslA3* | 391 | CslF | LOC_Os07g36700.1 | *OsCslF1* |
| 175 | CslA | LOC_Os06g42020.1 | *OsCslA9* | 392 | CslF | LOC_Os07g36740.1 | *OsCslF4* |
| 176 | CslA | LOC_Os07g43710.1 | *OsCslA7* | 393 | CslF | LOC_Os07g36750.1 | *OsCslF3* |
| 177 | CslA | LOC_Os08g33740.1 | *OsCslA11* | 394 | CslF | LOC_Os08g06380.1 | *OsCslF6* |
| 178 | CslA | LOC_Os10g26630.1 | *OsCslA2* | 395 | CslF | LOC_Os10g20260.1 | *OsCslF7* |
| 179 | CslA | PAXXG008150 | *PaCslA1* | 396 | CslG | AXF42_Ash021155 | *AsCslG1* |
| 180 | CslA | PAXXG029740 | *PaCslA2* | 397 | CslG | AXF42_Ash021157 | *AsCslG2* |
| 181 | CslA | PAXXG029760 | *PaCslA3* | 398 | CslG | AT4G23990.1 | *AtCslG3* |
| 182 | CslA | PAXXG185470 | *PaCslA4* | 399 | CslG | AT4G24000.1 | *AtCslG2* |
| 183 | CslA | PAXXG185480 | *PaCslA5* | 400 | CslG | AT4G24010.1 | *AtCslG1* |
| 184 | CslA | PAXXG238980 | *PaCslA6* | 401 | CslG | JL004975 | *CeCslG1* |

| | | | | | | | |
|---|---|---|---|---|---|---|---|
| 185 | CslA | LOC110018619 | *PeCslA1* | 402 | CslG | JL004976 | *CeCslG2* |
| 186 | CslA | LOC110018622 | *PeCslA2* | 403 | CslG | JL009243 | *CeCslG3* |
| 187 | CslA | LOC110021502 | *PeCslA3* | 404 | CslG | JL015126 | *CeCslG4* |
| 188 | CslA | LOC110021503 | *PeCslA4* | 405 | CslG | JL023141 | *CeCslG5* |
| 189 | CslA | LOC110025279 | *PeCslA5* | 406 | CslG | JL028218 | *CeCslG6* |
| 190 | CslA | LOC110028980 | *PeCslA6* | 407 | CslG | IEQ34_003039 | *DcCslG1* |
| 191 | CslA | HPP92_006012 | *VpCslA1* | 408 | CslG | IEQ34_003183 | *DcCslG2* |
| 192 | CslA | HPP92_006429 | *VpCslA2* | 409 | CslG | IEQ34_003185 | *DcCslG3* |
| 193 | CslA | HPP92_016790 | *VpCslA3* | 410 | CslG | IEQ34_003393 | *DcCslG4* |
| 194 | CslA | HPP92_016834 | *VpCslA4* | 411 | CslG | Dhu000010052 | *DhCslG1* |
| 195 | CslA | HPP92_027578 | *VpCslA5* | 412 | CslG | dof12478 | *DoCslG1* |
| 196 | CslA | HPP92_027583 | *VpCslA6* | 413 | CslG | dof12479 | *DoCslG2* |
| 197 | CslA | IEQ34_016385 | *DcCslA9* | 414 | CslG | dof12480 | *DoCslG3* |
| 198 | CslB | AT2G32530.1 | *AtCslB3* | 415 | CslG | dof12482 | *DoCslG4* |
| 199 | CslB | AT2G32540.1 | *AtCslB4* | 416 | CslG | dof12483 | *DoCslG5* |
| 200 | CslB | AT2G32620.1 | *AtCslB2* | 417 | CslG | dof12485 | *DoCslG6* |
| 201 | CslB | AT4G15290.1 | *AtCslB5* | 418 | CslG | dof12486 | *DoCslG7* |
| 202 | CslB | AT4G15320.1 | *AtCslB6* | 419 | CslG | PAXXG330010 | *PaCslG1* |
| 203 | CslB | JL001396 | *CeCslB1* | 420 | CslG | LOC110032318 | *PeCslG1* |
| 204 | CslB | JL023773 | *CeCslB2* | 421 | CslH | AXF42_Ash018798 | *AsCslH1* |
| 205 | CslB | JL025020 | *CeCslB3* | 422 | CslH | JL023774 | *CeCslH1* |
| 206 | CslB | JL026001 | *CeCslB4* | 423 | CslH | JL026003 | *CeCslH2* |
| 207 | CslB | LOC110038215 | *PeCslB1* | 424 | CslH | JL027031 | *CeCslH3* |
| 208 | CslB | HPP92_004406 | *VpCslB1* | 425 | CslH | IEQ34_018942 | *DcCslH1* |
| 209 | CslB | HPP92_027812 | *VpCslB2* | 426 | CslH | dof10594 | *DoCslH1* |
| 210 | CslB | AT2G32610.1 | *AtCslB1* | 427 | CslH | LOC_Os04g35020.1 | *OsCslH2* |
| 211 | CslC | AXF42_Ash000075 | *AsCslC1* | 428 | CslH | LOC_Os04g35030.1 | *OsCslH3* |
| 212 | CslC | AXF42_Ash000704 | *AsCslC2* | 429 | CslH | LOC_Os10g20090.1 | *OsCslH1* |
| 213 | CslC | AXF42_Ash007527 | *AsCslC3* | 430 | CslH | PAXXG233560 | *PaCslH1* |
| 214 | CslC | AXF42_Ash010865 | *AsCslC4* | 431 | CslH | PAXXG233600 | *PaCslH2* |
| 215 | CslC | AT2G24630.1 | *AtCslC8* | 432 | CslH | LOC110033336 | *PeCslH1* |
| 216 | CslC | AT3G07330.1 | *AtCslC6* | 433 | CslH | LOC110036772 | *PeCslH2* |
| 217 | CslC | AT3G28180.1 | *AtCslC4* | 434 | CslH | HPP92_004407 | *VpCslH1* |

**Supplementary Table 4. The number and distribution of motifs in eight subfamilies of nine orchid species.**

| Subfamily | Number of motifs | Present motifs | Absent motifs |
|---|---|---|---|
| cesA | 16 | motif 1-15,17 | motif 16,18,19,20 |
| cslA | 8 | motif 2,3,5,15,16,18,19,20 | motif 1,4,6,7,8,9,10,11,12,13,14,17 |
| cslB | 4 | motif 3,9,14,15 | motif 1,2,4,5,6,7,8,10,11,12,13,16,17,18,19,20 |
| cslC | 8 | motif 2,3,5,15,16,18,19,20 | motif 1,4,6,7,8,9,10,11,12,13,14,17 |
| cslD | 16 | motif 1-15,17 | motif 16,18,19,20 |
| cslE | 12 | motif 1-7,13-15 | motif 8,11,12,16,17,18,19,20 |
| cslG | 12 | motif 1-3,5,7,10-12 | motif 4,8,13,16,17,18,19,20 |
| cslH | 14 | motif 1-14 | motif 15,16,17,18,19,20 |

**Supplementary Table 5. Primer sequences of tested genes used in qRT-PCR.**

| NO. | Primer name | Primer sequence (5'to3') | Information for genes |
|---|---|---|---|
| 1 | *CeCslD1* F | GGCATCTACCCTTTCACCTCC | experimental |
| 2 | *CeCslD1* R | ACCACTCCTCAAGCCCTACG | experimental |
| 3 | *CeCslD8* F | GTCACCTTCCTCGCCTACCT | experimental |
| 4 | *CeCslD8* R | TCGTTTCGCCACCATTCCT | experimental |
| 5 | *CeCslA2* F | ACTCCAAGCTCATTCCACCTC | experimental |
| 6 | *CeCslA2* R | AACCCATTCGTTAACCCGAC | experimental |
| 7 | *CeCslA3* F | ATGATTCAACCGACCCCGA | experimental |
| 8 | *CeCslA3* R | CACCTGCTTTGTATCCCTTCC | experimental |
| 9 | *CeCesA2* F | TCCATTGAAGGAACCACCCC | experimental |
| 10 | *CeCesA2* R | GCTCCGTCATCAGACACATAACA | experimental |
| 11 | *CeCesA9* F | TTGTGGGAAGGAATAAGGAGG | experimental |
| 12 | *CeCesA9* R | GCCATTGGTGAGTAAAGGGAC | experimental |
| 13 | *Ce-TUB* F | GCAGTTTACGGCGATGTTCA | Reference for *Cymbidium ensifolium* |
| 14 | *Ce-TUB* R | ACTCTTCCTCGTCAGCTGTG | Reference for *Cymbidium ensifolium* |
| 15 | *GeCslD3* F | AACCCGTCTTCCACTGGTAAA | experimental |
| 16 | *GeCslD3* R | TCAGGCGTGAGAGGCATATC | experimental |
| 17 | *GeCslD4* F | GGAGGGAAACAATGGAAGCC | experimental |
| 18 | *GeCslD4* R | CCACGCCAGGAAGAAAAACA | experimental |
| 19 | *GeCslA1* F | TTCTTCGCCTTACCCCGTC | experimental |
| 20 | *GeCslA1* R | TCGCATTCCGTTCTCACCA | experimental |
| 21 | *GeCslA5* F | CCTTAAATCAGTAGGAACCCCA | experimental |
| 22 | *GeCslA5* R | GACAATCCATTCATTAGCCCG | experimental |
| 23 | *GeCesA2* F | CTATATGGTATGGGTATGGCGG | experimental |
| 24 | *GeCesA2* R | AAAGTGCAGTACAAGAGGAGCG | experimental |
| 25 | *GeCesA9* F | GCTATGTCCAGTTTCCGCAG | experimental |
| 26 | *GeCesA9* R | AATCCCATCAAGCCCTTTCA | experimental |
| 27 | *β-actin* F | GGGGATGAAGCACAGTCCAA | Reference for *Gastrodia elata* |
| 28 | *β-actin* R | GCCGTGGTTGTGAAGGAGTA | Reference for *Gastrodia elata* |
| 29 | *DoCslD6* F | GTCACATTCCTCACCTACCTCCT | experimental |
| 30 | *DoCslD6* R | CTGCTCATTTCGCCACCAT | experimental |
| 31 | *DoCslD12* F | TATGGGGTATGTCTGTAGTTTGCG | experimental |
| 32 | *DoCslD12* R | GGTGGGATTTGTAGGAGTGGG | experimental |
| 33 | *DoCslA2* F | CTTTGAAATAAGGGCGGGG | experimental |
| 34 | *DoCslA2* R | AGTGGGTCGTAAGGGAGGTG | experimental |
| 35 | *DoCslA10* F | CTCTCGATTGGGGCTTCGT | experimental |
| 36 | *DoCslA10* R | TCTTTCAGGGCACCTGCTTT | experimental |
| 37 | *DoCesA2* F | TTCTGTCTTTGTCGCTTCTACTCTT | experimental |
| 38 | *DoCesA2* R | TGCCTCCTTACCTCACTTCCC | experimental |
| 39 | *DoCesA6* F | TTGGTGTGGTTGCTGGGGT | experimental |
| 40 | *DoCesA6* R | TGGTCGGTGTTCTATTTTGTCG | experimental |
| 41 | *DoActin* F | TCAGGCTGACTGTGCTGTCCT | Reference for *Dendrobium catenatum* |
| 42 | *DoActin* R | GTGGTGGCGTCCATCTTGTT | Reference for *Dendrobium catenatum* |
